# Developing a More Rapid Test to Assess Sulfate Resistance of Hydraulic Cements

**DOI:** 10.6028/jres.110.080

**Published:** 2005-10-01

**Authors:** Chiara Ferraris, Paul Stutzman, Max Peltz, John Winpigler

**Affiliations:** National Institute of Standards and Technology, Gaithersburg, MD 20899

**Keywords:** cement paste, mortar, sulfate attack

## Abstract

External sulfate attack of concrete is a major problem that can appear in regions where concrete is exposed to soil or water containing sulfates, leading to softening and cracking of the concrete. Therefore, it is important that materials selection and proportioning of concrete in susceptible regions be carefully considered to resist sulfate attack. American Society for Testing Materials (ASTM) limits the tricalcium aluminate phase in cements when sulfate exposure is of concern. The hydration products of tricalcium aluminate react with the sulfates resulting in expansion and cracking. While ASTM standard tests are available to determine the susceptibility of cements to sulfate attack, these tests require at least 6 months and often up to a year to perform; a delay that hinders development of new cements. This paper presents a new method for testing cement resistance to sulfate attack that is three to five times faster than the current ASTM tests. Development of the procedure was based upon insights on the degradation process by petrographic examination of sulfate-exposed specimens over time. Also key to the development was the use of smaller samples and tighter environmental control.

## 1. Introduction

External sulfate attack of concrete can adversely affect a concrete structure by softening and cracking the concrete. This phenomenon is more prevalent in arid regions and in areas where ground waters and soils contain sulfates either naturally or through industrial exposure. Current ASTM International predictive tests measure mortar bar expansion under sulfate exposure, setting limits on expansion to assess whether the corresponding concrete will be sulfate resistant (ASTM C 1012) [1]. These tests usually require measurements that last from 6 months to a year, which is a long time for a cement manufacturer to assess new hydraulic cements. Therefore, there is a need for a new accelerated test. In this paper, we will critically examine standard test methods, discuss the basics of the chemical interaction between the cement and the sulfate, and provide detailed information on the new method proposed.

## 2. Background

### 2.1. Test Methods

ASTM International^1^ provides material specifications and standard test methods for construction materials. A prescriptive specification describes a product predominantly by its composition, while a performance specification describes how well a product must perform. ASTM C 150 [2] and ASTM C 595 [3] are essentially prescriptive standards for cement and blended cement, while a performance specification covering all hydraulic cements is described in ASTM C 1157 [4]. An example of the difference between prescriptive and performance requirements is found in comparing ASTM C 150 and ASTM C 1157. ASTM C 150 limits tricalcium aluminate^2^ (C_3_A) levels for Type II and V portland cements of 8 % and 5 %, respectively, in order to control sulfate resistance. The performance-based ASTM C 1157 utilizes a physical test (ASTM C 1012) for sulfate resistance by evaluating expansion of mortar prisms made with the cement and requiring them to have expansion below a certain limit without specifying the cement composition limits.

Thus, critical issues with regard to performance standards are test methods and criteria for evaluation of performance. However, durability testing is difficult as most concrete products are expected to last decades. Since there are few long-term tests underway today, accelerated tests must be developed and the properties being measured in the laboratory must correspond to field performance. A change to performance-based standards could dramatically impact the cement and concrete industry. Without prescriptive composition limits, cements likely would be manufactured over a wider range of chemical and physical characteristics, allowing more efficient use of raw materials and reduced energy consumption and environmental emissions. While cement strengths would remain a major factor in user purchase decisions, other performance characteristics, such as durability, could assume greater significance [5,6].

In a project co-sponsored by the Portland Cement Association (PCA), the mechanisms leading to sulfate attack in a concrete structure were examined. They were divided into two categories: 1) the penetration of the sulfate through sorption or diffusion [7]; and 2) the chemical reactions between the cement and its hydration products and the sulfate ions. The first category resulted in the development of a new ASTM standard, C 1585 [8], which measures sorption characteristics of concrete and has stimulated discussion on how to measure diffusion coefficients in cementitious materials [9]. Currently, the industry uses ASTM C 1202 [10] (the so-called Rapid Chloride Test) to estimate the penetration of ions in a fully-saturated sample. However, this method does not determine diffusion coefficients, but provides a comparative measure of the resistance of chloride ions, diffusion of concrete specimens. As regards chemical reactions, the only test that indirectly determines the resistance of a cement to sulfate is ASTM C 1012 through measuring the expansion of a specimen immersed in a sulfate solution (usually sodium sulfate). This test requires measurements for 6 months to a year.

ASTM C 1012 consists of preparing three prisms (25.4 mm × 25.4 mm × 279.4 mm) of mortar with a special pin cast in both ends of the specimen to facilitate length change measurement. The specimens are cured in limewater until they achieve a strength of 20 MPa, and are then placed in the sodium sulfate solution. The change in length is monitored using a frame and a comparator at designated test times. The cement is considered sulfate resistant if the expansion does not exceed a pre-selected value, most often 0.1 %, after 6 months or a year.

In this study, a sulfate attack performance-based methodology was examined to gain a better understanding of the effects of testing on mortar bar microstructures. Petrographic characterization of the microstructural changes over time was documented. The measurements on mortar bars were done following the methodology described in ASTM C 1012, as this is the standard test used in ASTM C 1157. These data obtained in this study provide insights about the processes involved in the alteration of the hardened cement constituents.

Therefore, after close examination of the kinetics of the chemical deterioration and a critical evaluation of ASTM C 1012, a new method based on smaller specimens has been developed. Validation testing demonstrates a reduction in testing time by a factor of three to five. We show that an improved understanding of the deterioration process may allow development of an ever more rapid performance test procedure.

### 2.2 The Chemical Mechanisms

Skalny and Pierce summarized current knowledge on sulfate attack [11], that it “is a complex sequence of physical and chemical processes resulting in chemical and physical (micro-structural) modifications of the cement paste matrix”, leading to the “loss of mechanical and physical properties of a structure.” The confounding effects of cement composition, concrete mixture design, chemical and mineral admixtures, concrete placing and curing procedures, and complicated (often variable) environmental exposures make it quite difficult to sort out key factors in the process of sulfate attack and to evaluate their effects.

Brown and Taylor [12] outlined the following sequence of events during interaction of Na_2_SO_4_ solutions with hardened cement pastes, mortars, and concretes. Initially, ettringite is formed with aluminate ions being supplied by monosulfate and un-reacted C_3_A. In addition, a source of calcium is needed, which may be found from the dissolution of calcium hydroxide and later decalcification of the calcium-silicate-hydrate (C-S-H). This continues as long as the pH is within a range where ettringite is stable (10.7 to 12.5) [7]. Close to the surface of the specimen, the sulfate concentration in the pore solution is relatively high, leading to the precipitation of gypsum.

## 3. Experimental Testing

### 3.1 Materials

Specimens were prepared using cement and one supplementary cementitious material from the larger set [13]. The cement chemical compositions shown in Table 1, and Table 2 shows estimates of the mineralogical composition based either on ASTM C 150 or on x-ray diffraction. XRD data represent averaged values from a bulk sample and a salicylic acid/methanol extraction residue.

One supplementary cementitious material (SCM) was used, a Class C fly ash (FA-C). The dosages were set to be 15 % by mass fraction of cement replacement for SCM. This SCM was selected because FA-C replacement cement results in an expansion faster than the control. Further details on the materials and additional tests are presented in Ref. [13].

Three specimen geometries were prepared: 1) small prisms (10 mm × 10 mm × 40 mm) of cement paste, and 2) small prisms (10 mm × 10 mm × 40 mm) of mortar: and 3) standard prisms as described in ASTM C 1012. All specimens were cured in limewater for 7 d before exposure to sulfate solution. See Sec. 4 for the results obtained and further discussion.

### 3.2 Microstructure Observations on Test Specimens

Specimens that fail the ASTM C 1012 test often exhibit a consistent length change followed by a rapid increase in length. Additionally, as testing proceeds, the surfaces spall, exposing deeper regions of the mortar bar. Tracking changes in the mortar microstructure against expansion may provide better insight on the effects of the test method’s exposure conditions.

Alteration effects are seen after a few days’ exposure of cement paste to sulfate solutions [50 g/L Na_2_SO_4_ (the same solution as in ASTM C 1012)] with the replacement of CH by gypsum in the outer portions of the cement paste. However, this replacement does not appear to be disruptive. This replacement forms a front that migrates inward with time and progresses to a depth of a few millimeters. Eventually, three zones outside of the apparently unaltered cement paste become distinct: 1) An outer zone leached of most constituents (including ettringite), leaving a more porous, calcium-depleted CSH and some remnant ferrite; 2) A second zone where gypsum replaces calcium hydroxide and ettringite replaces monosulfate; and 3) A third zone with monosulfate that appears to be sulfur-rich or perhaps a mixture of ettringite and monosulfate. At later stages of testing, specimens exhibit cracking parallel to the exterior in the outer zones, with the fractures generally filled by gypsum.

**Fig. 1:**
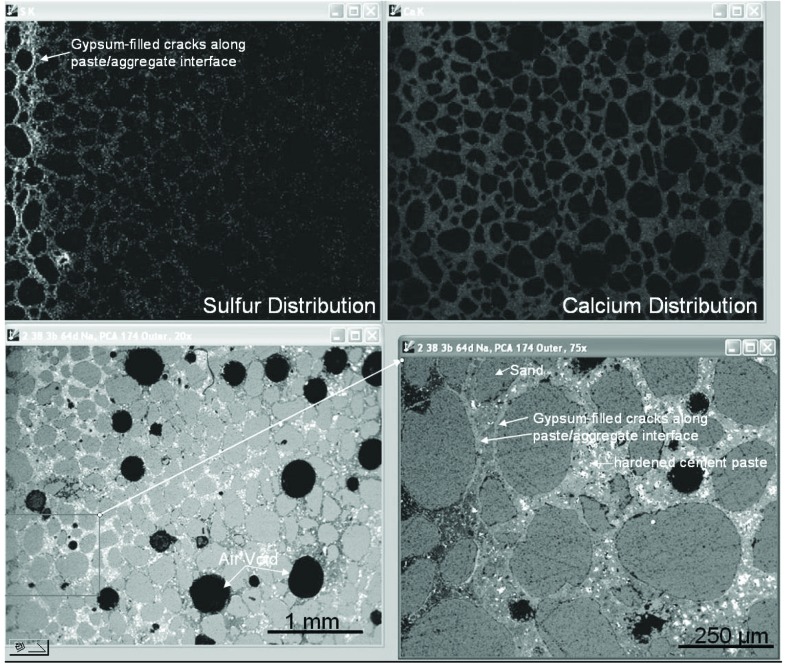
After 64 d of exposure, backscattered electron images at low (lower left) and higher (lower right) and x-ray images for sulfur (upper left), and calcium (upper right) show the cracking below the surface and gypsum (CaSO_4_·2H_2_O) filling cracks. The surface is oriented toward the left. (Scale indicated in the figures)

Figure 1 shows a wide-range view (about 4 mm) of the mortar bar with the side surface oriented to the left. Cracking parallel to the surface is highlighted by the gypsum-filled cracks demonstrated by the high sulfur regions of the x-ray image (upper left) and the lighter phase rimming the sand grains in the lower-right higher-magnification image. The presence of gaps along aggregate boundaries is an indication that the hardened cement paste matrix has expanded. The slight decrease in overall intensity in the calcium image and backscattered electron (BSE) image reflects the calcium depleted outer zone. The gypsum is generally confined to this outer portion of the mortar, and microanalysis evidence indicates changes in monosulfate and calcium aluminate phases at greater depths that probably are contributing to the expansion.

**Fig. 2:**
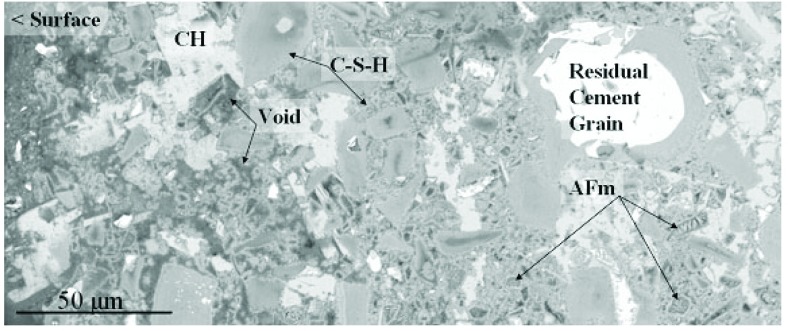
Cross-section of hardened cement paste, not exposed to sulfate solution, showing residual cement grains, calcium hydroxide (CH), calcium-silicate-hydrate (CSH), monosulfate (AFm), and voids.

Figure 2 illustrates a higher-magnification, cross-sectional view of a hardened cement paste in a lime water-cured, control mortar specimen. The outer surface is oriented to the left, with a total field width of 250 μm. The residual cement grains appear brightest followed by calcium hydroxide (CH), calcium-silicate-hydrate (C-S-H) and dark voids that are filled with epoxy. The C-S-H may be further subdivided into outer-C-S-H, formed in the originally water-filled spaces, and inner-C-S-H, formed by in-situ hydration of the cement grains. The outer-C-S-H has a coarser porosity and so appears slightly darker. Other constituents such as ettringite (AFt) and monosulfate (AFm) occur in the bulk paste and may be identified based upon their textures and chemical signatures.

**Fig. 3:**
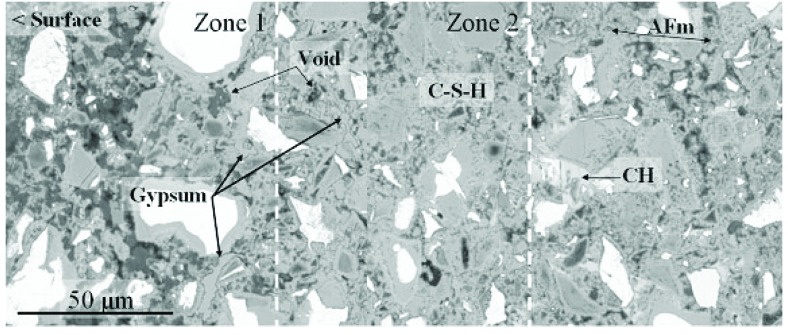
Type I/II cement paste (105 d of exposure to sulfate solution), Na_2_SO_4_-soaked specimen, showing increased porosity near the surface (left, zone 1), loss of calcium hydroxide in outer 150 μm (zones 1 & 2), possible densification of inner-product CSH in CH-depleted zone 2, deposition of gypsum in place of CH, and replacement of monosulfate with ettringite.

Figure 3 shows a cross-section of hardened cement paste (250 μm field width) that has been exposed to the sodium sulfate solution for 105 d, exhibiting 0.14 % expansion. Changes in microstructure relative to the control include an increased porosity near the surface, and a loss of CH within 150 μm of the surface. A second zone may be characterized by replacement of monosulfate with ettringite, a densification of inner-product C-S-H (seen as a loss of coarse-capillary porosity), and deposition of gypsum in place of CH. At greater depths, the spot chemical analysis indicates increased sulfate content in the C-S-H relative to the control and mixtures of monosulfate and ettringite in regions formerly occupied by monosulfate. The first two zones can be seen to migrate inward over testing time but the gypsum remains within a few millimeters of the surface.

Clifton and Pommersheim [14] examined the potential volume change associated with selected chemical reactions in concrete. They calculate that for reactions involving sulfates with monosulfate, significant increases (129 %) could be expected if the sulfate was in solution. Given that monosulfate volume fraction is generally in the 10 % to 15 % range for hydrated cement, this creates a potential for expansion.

**Fig. 4:**
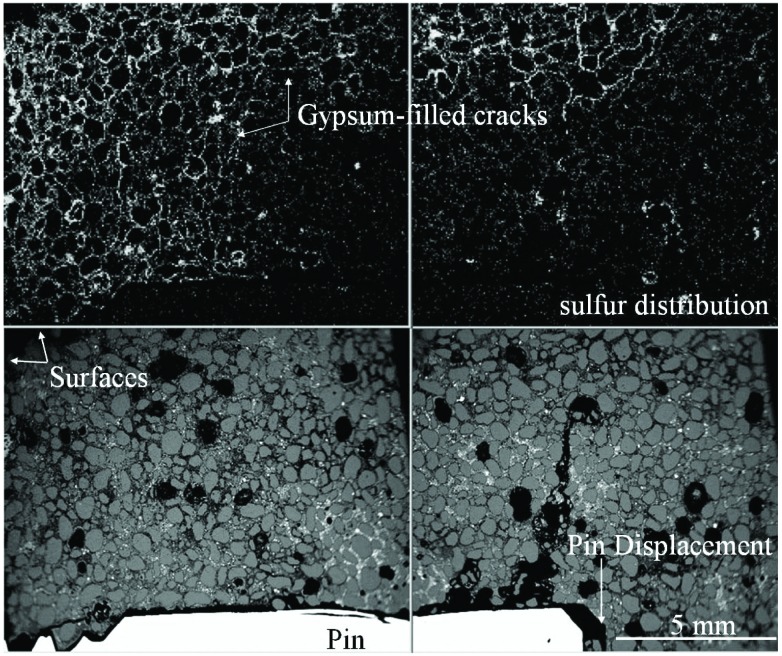
Composite image with about 12 mm total field width with the backscattered electron image of the mortar and end pin (bright rod) and above x-ray images of sulfur highlighting gypsum-filled cracks in the mortar. The gap at the base of the pin is about 0.5 mm in width.

As noted earlier, one characteristic for cements that fail the test is a rapid increase in expansion. Imaging a specimen after this increase shows a number of interesting phenomena that may explain its cause. A composite image (Fig. 4, about 12 mm total field width) of the BSE image of the mortar (bottom) and x-ray images of sulfur (top) with the mortar bar end oriented to the left showing the measuring pin as the brightest object in the BSE image. Two features are evident: 1) the gypsum-rich regions extend deeper into the bar at the ends relative to the central part of the bar and 2) the expansive reactions in the end have lifted the measurement pin about 0.50 mm out of its socket.

The sulfate solution has penetrated from both the end and side surfaces of the bar. This penetration from multiple directions results in the bar ends being more completely affected than the mid-length portions of the bar (Fig. 3). The highly affected area (as defined by gypsum-filled cracking parallel to the surfaces) extends about half the length of the pin, which is then lifted by the expansion of the outer mortar. This would indicate that the early expansion measured is not the result of the entire cross section reacting to the sulfate infiltration and that the core, up to that point, has served to restrain expansion. The measurement of expansion according to ASTM C 1012 is based on the assumption that the whole specimen is expanding at the same time, while what we observed is that only a small fraction around the pin is responsible for the bulk of the measured expansion. Therefore, given this specimen configuration and lack of protection of the end portions, only a fraction of the whole standard specimen is really affected by the sulfate. As expansion is measured as a percentage of the total length affected, the *real* expansion near the pin could be 50 times larger than reported. By *real* expansion, we mean the change in length due to the chemical reaction divided by the length of affected specimen.

Therefore, two solutions to the deterioration around the pin and in the outer layer are proposed: 1) protect the ends of the specimens so that the sulfate penetration cannot occur from these surfaces and around the pin; 2) reduce the cross-section of the specimens to shorten the time necessary to permeate the specimen with the sulfate. The combination of these modifications will protect the end surfaces and pin region from sulfate penetration while decreasing the time for the solution to permeate the test bar cross section. This should produce a more effective configuration to measure expansion of the whole specimen (not just the outer layer or around the pins).

### 3.3 Experimental Set-Up for the Proposed Method

**Fig. 5:**
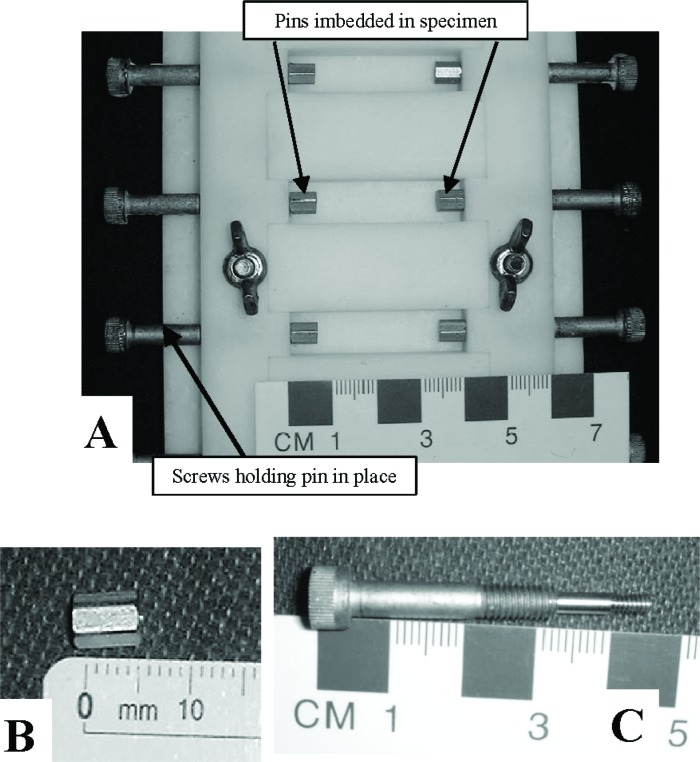
Molds for specimens: a) general view; B) Pin imbedded in the specimen; C) Screw to hold pins in place during cast.

Molds were custom designed and machined from a block of Teflon^3^ to produce specimens 10 mm × 10 mm × 40 mm with pins imbedded in both ends (Fig. 5A). The pins used are threaded standoff (4/40 thread, 4.76 mm (3/16 in) O.D., 6.35 mm (1/4 in) length) (Fig. 5B). The pins are held in place during the sample preparation and curing by a modified screw (Fig. 5C). Since the total volume of materials is substantially less than that required in ASTM C 1012, modified mixing, placing and consolidating procedures were developed.

The mixing procedure consists of mixing together the cement and the water using a speed-controlled blender with a 250 mL beaker. The cement was introduced in 30 s to the water while mixing at about 419 rad/s (4000 rpm). With the mixer maintaining the speed of 419 rad/s, the cement paste was mixed for another 30 s. After 2.5 min of rest, the cement paste was mixed at 1047 rad/s (10 000 rpm) for 30 s.

To prepare the specimens, the cement paste is placed in the molds and compacted by small taps on the side of the mold. Care is taken to ensure that the cement paste surrounds the pin imbedded in the sample. The mold is placed in a closed plastic bag with some water to maintain 100 % RH and the bag is stored for 24 h in a curing cabinet at a constant temperature of 22 °C ± 2 °C. After 24 h, the specimens are demolded and placed in limewater. The container with the specimens in limewater is placed in the same curing cabinet to maintain constant temperature.

**Fig. 6:**
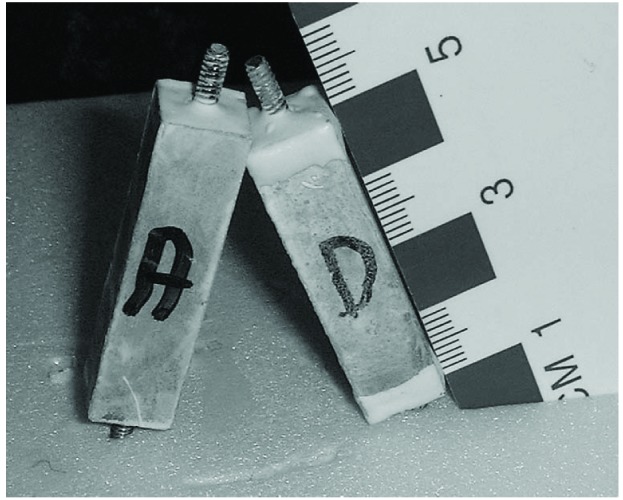
Specimens curing with epoxy coated ends; Specimen D was coated also on 5 mm on the side; Specimen A had only the ends sections coated. It was determined that the D type coating give more reproducible results [13].

**Fig. 7:**
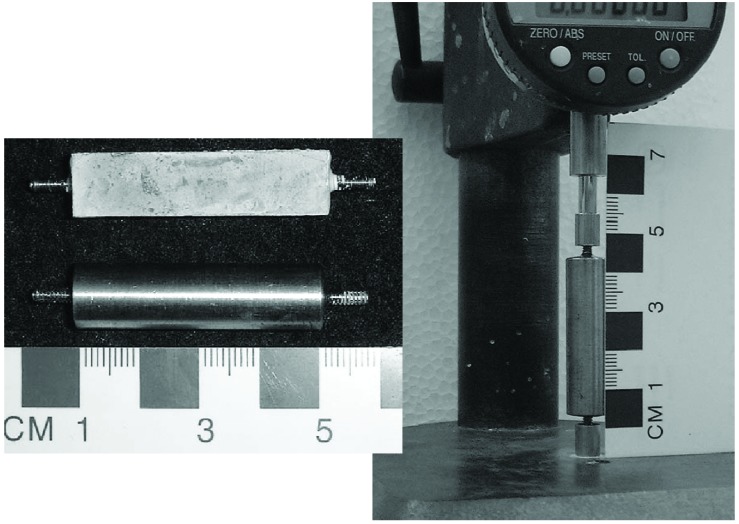
Expansion measurement: A) reference and a specimen; B) comparator with the reference in place.

Three or four days after mixing, the specimens are removed from limewater and a threaded stud is screwed in the end pins (Fig. 6 and Fig. 7A). The threaded stud is 12.7 mm long and has the same thread as the pin (4/40). To ensure that the stud does not move during the expansion experiment a small amount of epoxy is placed around one end of the studs and threaded into the specimen ends. Epoxy is applied to both end faces around the studs and 5 mm on the top side of the specimen to prevent sulfate penetration from the ends (Fig. 6). The epoxy is cured by leaving the specimens in a 100 % RH for about 5 h to 6 h. Water should not contact the specimen or the epoxy during this curing. The specimens are then returned to the limewater until the start of exposure to sulfate solution, usually 7 d after casting. During exposure to sulfate, the specimens are kept in a temperature controlled cabinet at 23 °C ± 0.5 °C.

A length change comparator (Fig. 7B) is used to measure expansion, with a stainless steel or, if possible, Invar cylinder (Fig. 7A) as a reference length to zero the comparator. The pins for the comparator are custom fabricated to accommodate the studs of the specimens.

After curing, the samples are measured and placed in a sulfate solution 50 g/L Na_2_SO_4_ (the same solution as in ASTM C 1012). It is strongly suggested that the container with the specimens be kept at constant temperature to reduce length changes from temperature fluctuations. The controlled temperature reduces the scatter significantly, especially during the first days [13]. The specimens are then measured every day for the first 2 weeks and then once a week until deterioration commences.

## 4. Results and Discussion

The goals of the test program for measuring expansion were to: 1) check that the expansion could be measured using small specimens; 2) determine the reproducibility of the measurements; 3) compare the results with those obtained with the larger (ASTM C 1012) specimens. In all cases, the cement performance was compared with cement with addition of SCM.

### 4.1 Small Prisms of Cement Paste or Mortar

**Fig. 8:**
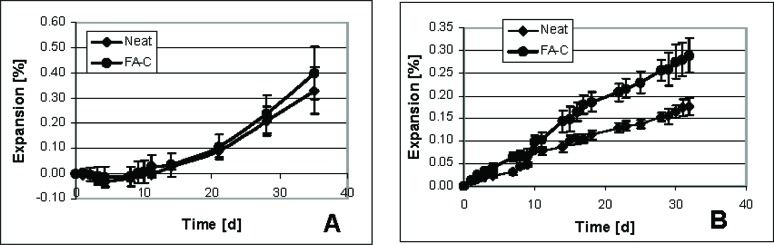
Average expansion of small sample versus time: A) mortar samples; B) cement paste samples. The standard deviations of the five specimens are shown as uncertainty bars.

Two sets of small specimens were prepared: 1) mortars and 2) neat cement paste. Distinct differences emerged upon comparing mortar and paste-only specimens (Fig. 8) with mortar specimens showing greater expansion than the corresponding cement pastes. This could be explained by the faster penetration of the sulfate through the more permeable mortar. However, the mortar specimens do not show a difference in expansion between the two cements (FA-C and control), while there is a significant difference after 20 d for the cement paste specimens. After 40 d of measurement, the mortar and the cement paste specimens were “no longer measurable”. The definition of “no longer measurable” indicates that either the sample is too long for the comparator, or the sample is in very poor state or disintegrated.

Repeatability in mortars is poorer than that for the cement paste as seen in the larger uncertainty bar widths. Intrinsically mortar specimens have a more random microstructure due to the random aggregate structure superimposed on the cement paste microstructure. Also, the difficulty in casting and consolidating small mortar specimens, especially in consolidation around the pin area lessens the repeatability. No problems with casting and consolidation were observed using neat cement paste, and elimination of the sand reduces preparation efforts. Therefore, the cement paste specimens appear to provide faster and more reliable results than the mortar specimens.

The results presented in Fig. 8 are from five specimens for each composition. The selection of the five replicates is somewhat arbitrary and further studies could be conducted to determine if the number can be reduced. The risk of selecting fewer specimens is potentially higher scatter in the results.

### 4.2 Larger Prisms According to ASTM C 1012 and Comparison With Small Prisms

**Fig. 9:**
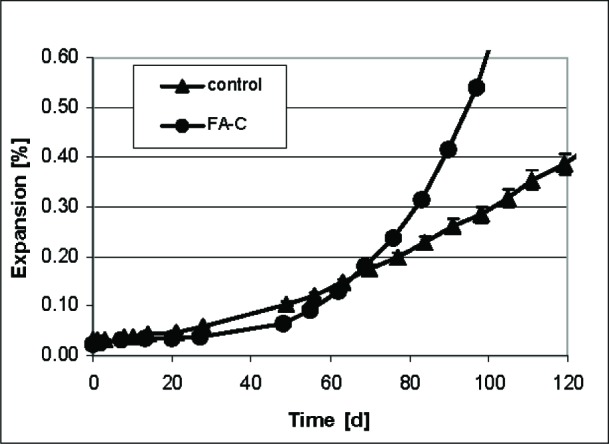
Average expansion of large specimens versus time. The standard deviations of the three specimens are shown as uncertainty bars. For FA-C, the uncertainty bars are smaller than the data symbol.

Figure 9 shows results obtained using the standard prisms specimens in ASTM C 1012 (25.4 mm × 25.4 mm × 279 mm). The ranking of the resistance to sulfate attack is the same as for the smaller cement paste samples, e.g., the specimens with Class C fly ash expand more than the controls during the time of the measurements.

As is done in ASTM C 1012 [2,3], the time to reach an expansion of 0.1 % was taken as the critical time to classify the performance of the cements. Table 3 shows the results. The small cement paste specimens ranked the cements in about 20 d to 30 d, while the large specimen required twice the time while ranking the control as worse or identical to the cement with fly ash. If the testing stopped at this point, the conclusions reached with the large prisms would not be a valid assessment of the resistance of these cements, as it is known that addition of FA-C is deleterious for sulfate resistance of cement paste. In Table 3 we also show the expansion reached at an arbitrary later date, 30 d for small specimens and 100 d for the larger specimens. We could not select the same date for both types of specimens because 1) the large specimens at 30 d were below 0.1 % expansion; 2) the small specimens were not longer measurable after 30 d and 3) at 120 d the FA-C large specimen were not longer measurable. Therefore, from Figs. 8B and 9 and from Table 2, it could be inferred that the FA-C cement is less sulfate resistant than the control. The results for small specimens mirror those for larger specimens and provide a result in at least a third to a fifth of the time for the cements shown here that were highly reactive. A similar reduction was seen in preliminary tests (not shown here) for cements that were not highly reactive, including hydraulic cements conforming to ASTM C 1157 [4].

Obviously, these preliminary observations of the relative behavior of the two types of specimen should be verified with further testing of various cements.

## 5. Conclusion

Rapid standard tests are necessary in developing new hydraulic cements for construction. This paper presents a new and more rapid method based on material science to measure expansion of the cement paste exposed to a sulfate solution. This method is based on petrographic examination specimens exposed to sulfate and uses smaller test specimens than those utilized in the current standard tests (ASTM C 1012). The same conclusions on the potential sulfate resistance of a cement can be obtained using either method, but the new method described here requires less than one third the testing time compared to ASTM C 1012.

This study exposed some problems inherent with the current test method described in ASTM C 1012. Permeation of the test solution occurs along the mortar bar ends, sides and pins resulting in the measurement of reaction and expansion of only a portion of the test specimen. If this issue was corrected the standard specimens will likely take even longer to expand to the deleterious level. By using a test specimen with a smaller cross-section and protected ends we were able to reduce testing time and measure the expansion due to sulfate interaction with cement paste hydration products across the entire test specimen. Temperature control becomes paramount when small samples are used and help reduce scatter of the data especially at early age of the testing.

This study presented only some of the data available and it needs to be further validated by conducting tests on a larger selection of cements.

## Figures and Tables

**Table 1:** Cement composition

	Type I/II Mass fraction %
SiO_2_	20.4
Al_2_O_3_	4.39
Fe_2_O_3_	2.64
CaO	63.6
MgO	4.21
SO_3_	2.76
LOI	0.53
Na_2_O	0.156
K_2_O	0.48
TiO_2_	0.440
P_2_O_5_	0.081
ZnO	0.04
Mn_2_O_3_	0.09
Cl	0.010
V_2_O_5_	0.01
Cr2O_3_	0.02
NiO	0.01
SrO	0.04
ZrO_2_	0.02
BaO	0.07

**Table 2:** Compositional estimates

	Type I/II Mass fraction %	
	ASTM C 150	XRD
Alite	62.5	62.1
Belite	11.4	12.1
Aluminate	7.2	4.5
Ferrite	8.0	9.7
Arcanite		0.4
Periclase		2.3
Gypsum		0.7
Bassanite		3.1
Anhydrite		0.3
Dolomite		0.8
Calcite		0.5
Quartz		0.2

**Table 3:** Time in days to reach 0.1 % expansion and expansion at an arbitrary date

	Time in days to reach 0.1 % expansion		Expansion	
	Small sample method (d)	ASTM C 1012 (d)	Small sample at 30 d (%)	ASTM C 1012 at 100 d (%)
Control	15	49	0.2	0.3
Class C Fly ash	10	55	0.3	0.7

## Footnotes

1 http://www.astm.org

2 The cement chemistry notation is used for the rest of the paper: C = CaO; H = H_2_O; S = SiO_2_; A = Al_2_O_3_.

3 Certain commercial equipment, instruments, or materials are identified in this paper to foster understanding. Such identification does not imply recommendation or endorsement by the National Institute of Standards and Technology, nor does it imply that the materials or equipment identified are necessarily the best available for the purpose.
